# Teaching From Afar: Development of a Telemedicine Curriculum for Healthcare Workers in Global Settings

**DOI:** 10.7759/cureus.20123

**Published:** 2021-12-02

**Authors:** Jason T Lowe, Sunny R Patel, Wei D Hao, Jamie Johnston, Abdullah Butt, Matthew Strehlow, Benjamin Lindquist

**Affiliations:** 1 Emergency Medicine/Pediatric Emergency Medicine, Stanford University School of Medicine, Lucile Packard Children's Hospital Stanford, Palo Alto, USA; 2 Emergency Medicine, Weill Cornell Medicine, New York, USA; 3 Emergency Medicine, Stanford University School of Medicine, Palo Alto, USA; 4 Stanford Center for Health Education, Stanford University School of Medicine, Palo Alto, USA; 5 eDoctor, EduCast, Karachi, PAK

**Keywords:** telehealth education, telehealth, virtual visits, video telemedicine, covid-19, international medicine, telemedicine education, education, telemedicine

## Abstract

The Stanford Department of Emergency Medicine joined forces with Digital Medic to create educational materials to teach global healthcare providers how to evaluate patients via telemedicine in the setting of COVID-19. Users then asked for additional education on best practices surrounding the use of telemedicine as a communication medium. Here, we describe our experience in the creation of this additional module and provide some basic feedback received from end-users. We scripted, filmed, and edited a video module for this application over the course of 14 weeks. It was subsequently deployed as part of the larger COVID-19 educational program. To date, the course has had over 28,000 participants. Each was asked to take a pre- and post-test to assess the knowledge of telemedicine best practices before and after the video module; 19,412 elected to take the pre-test and 19,364 took the post-test with overall scores of 84% and 95%, respectively. Anecdotal feedback has been positive. Telemedicine systems have proliferated rapidly around the world, but best practices for physician-to-patient interactions have not been similarly disseminated. We conclude that video modules can be used to fill this educational need quickly and economically.

## Introduction

The COVID-19 pandemic impacted all aspects of human existence, regardless of culture or geographic location. Traditional delivery of healthcare services utilizing in-person visits rapidly declined as the world focused on the moderation of high-risk contacts and reduction of personal protection equipment (PPE) use. Telemedicine and virtual visits increasingly became the media of choice for patients and healthcare workers (HCWs) to interface in a low-risk manner. 

In the spring of 2020, as the COVID-19 pandemic continued, our emergency department collaborated with Digital Medic (https://digitalmedic.stanford.edu/) to create a series of educational videos for global HCWs (https://online.stanford.edu/courses/som-xche0007-covid-19-training-healthcare-workers). This course focused on the evaluation and treatment of patients with COVID-19 and was delivered free of charge through various open-access educational platforms. 

Subsequently, by request, our physician group collaborated with EduCast (https://edu.educastportal.com/), an educational organization providing telemedicine services in Pakistan, to create specific video content on best practices for the conduction of telemedicine visits. The video module we created was then released to all course participants. In this article, we have reported the development of this educational program and the initial reception from our learners, including knowledge gained from the intervention. Ethical approval for this study was obtained from the Institutional Review Board from Stanford University (IRB No.: 57831).

## Technical report

Phase 1: Review and assessment of current educational curriculum for virtual visits 

Eight weeks after the deployment of the global COVID-19 course, the EduCast physicians remotely caring for COVID-19 patients in Pakistan returned feedback that additional training on the specifics of conducting a telemedicine visit would be a desirable addition to the educational offerings. Before embarking on creating materials de novo, our group performed a literature review on PubMed and Google search (“telemedicine best practices,” “telehealth best practices,” and “how to conduct a telemedicine visit”) to review the previously created materials. The search identified limited current literature on telemedicine best practices. A white paper from the American College of Emergency Physicians from 2018 provided an overview of the state of telehealth and gap areas. Of note, it did not specifically address telemedicine as a medium to reduce high-risk interactions [1]. Other identified publications did not address direct physician-to-patient interactions or focused on policy or broader institutional concerns [2-4]. We aimed to design a succinct course focused on teaching the very basics of a virtual visit in the setting of COVID-19 for low-resource settings.

Phase 2: Development of educational content

The Digital Health Team of the Stanford Department of Emergency Medicine was asked to create telemedicine education videos due to our experience with the development of similar educational materials for our institution's telemedicine program. We broadly divided topics into technical and clinical sections based on our prior experience and target audience. From there, our final module covered the subject areas seen in Table 1.

**Table 1:** Telemedicine education topics

Learning Topics	Length (minutes)
Introduction	1
Setting Up Virtual Visits	3
Conducting a History	2
Acquiring Vital Signs	1
Completing a Physical Exam	2.5
Tips for Managing COVID-19	1

**Figure 1:**
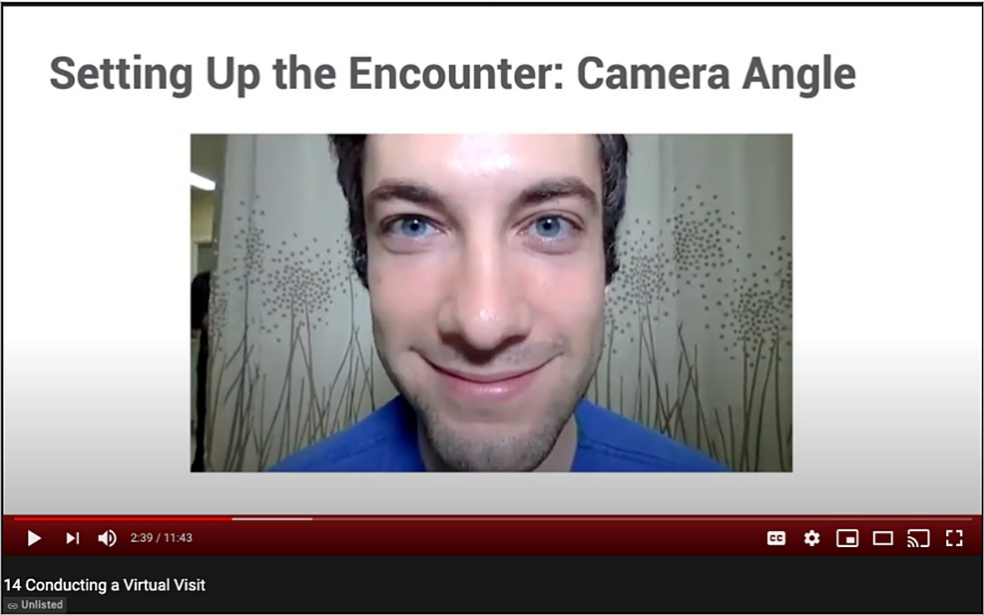
Sample frame of telemedicine video module

Technical considerations included camera placement, lighting placement, and audio considerations. Though many users may have familiarity with FaceTime and other consumer-to-consumer media, switching to a professional, clinical environment necessitates purposeful consideration. The camera should be placed such that the provider’s image is centered on the screen, with the provider looking into the camera to maintain the semblance of eye contact. Most built-in phone or laptop cameras have small sensors, so maximizing ambient light is necessary to provide the sharpest, clearest image. It is also important to avoid backlighting that creates a shadow over the face (Figure 1). Further, we emphasized minimizing extraneous background noise to improve the audio quality.

Clinical topics of discussion followed traditional history and physical exam workflows such as head, eyes, ears, nose, and throat (HEENT). In this setting, providers did not have the use of peripheral medical equipment (e.g., stethoscope or otoscope), so maximizing the information available was paramount. Pulmonary exams, without auscultation, included assessment of work of breathing and the ability to speak with a patent airway and in full sentences without developing breathlessness. The cardiac module focused on skin color and instructed the patient to perform a distal perfusion exam on themselves. 

We also included a section specifically aimed at helping HCWs to identify high-risk patients in the setting of COVID-19. These included patients at the extremes of age and those with pre-existing comorbidities. Once again, a special emphasis was placed on the respiratory status by highlighting an observational respiratory evaluation.

Once topics of discussion were delineated, a detailed script was written. All scenarios and lines were reviewed by several of the authors (JL, DH, SP, BL). Once the script was approved, five actors from our emergency medicine residency program were recruited to perform designated roles, including healthcare providers and patients. 

Filming occurred in a repurposed section of our emergency department that does not serve as a patient care area. The total time filming on set was two hours. We solicited the aid of our in-house audiovisual assistant to set up cameras and microphones. Video equipment used included a Sony ZV-1 consumer camera and a Sony A7RIII prosumer camera (Sony Corp., Tokyo, Japan) with 70-200 mm f.2.8 lens and 50-mm f.1.8 lenses. The audio was recorded using onboard camera microphones in addition to a Tascam DR-10L microphone. Lighting was provided by a Neewer LED video-lighting kit.

To minimize direct contact, post-production voiceover work was recorded virtually on Zoom using a Blue Yeti USB microphone and a Blue Nano USB microphone. Post-production editing was performed on a 2019 Macbook Pro running Adobe Premiere video-editing software. Graphics were created on Adobe Illustrator and After Effects by Digital Medic, who also edited and produced the final video.

From project conception on September 2, 2020, to the final release on December 15, 2020, the time course was 14 weeks. All work was done on a volunteer basis. The final educational video was 11:43 minutes in length.

Phase 3: User feedback and further planning

Since the deployment of the module on September 20, 2020, over 28,000 participants have taken the course. A pre- and post-test covering all modules was administered. The pre-test had 22 questions totally with two based specifically on our telemedicine module. The post-test had 24 questions totally with four based on our telemedicine module (Table 2); 19,412 elected to take the pre-test, and 19,364 elected to take the post-test. We found that the overall scores improved with an average pre-test average score of 81% on the first attempt and an average post-test score of 86% on the first attempt. See Table 3 for additional score breakdown by question.

**Table 2:** Pre-test (questions 1-2) and post-test (questions 1-4)

Lecture: Conducting a Virtual Visit	Answers
1. You are asked to care for a patient via telemedicine. The patient is a 64-year-old male with a history of diabetes and hypertension who is concerned about having COVID-19. Which of the following patient complaints may require in-person evaluation?	a. Cough
	b. Fever
	c. Headache
	d. Shortness of breath
2. You are caring for a 25-year-old male via a telemedicine encounter. You notice that your face is quite dark on the screen. Where can you position the light source in your room to improve visualization?	a. Directly behind your head facing forward
	b. Directly in front of you facing your face
	c. Directly to the left side
	d. Directly to the right side
3. You are caring for a 35-year-old male via telemedicine. He likely has COVID-19 and has been having symptoms for 7 days. He reports increasing levels of shortness of breath and fatigue. He looks quite dyspneic over the computer screen. What are the next steps for this patient?	a. Arrange transport to the hospital
	b. Prescribe Azithromycin for possible pneumonia
	c. Prescribe Dexamethasone for possible inflammatory reaction
	d. Schedule repeat telemedicine visit for the following day
4. You are caring for a 68-year-old female via telehealth and are attempting to calculate her respiratory rate. Which of the following options will give you the most accurate rate?	a. Inform the patient that you need to count the number of breaths
	b. Inform the patient that you need to observe their breathing and to breathe normally, then begin counting each breath for 15 seconds and multiplying by four
	c. Inform the patient to sit quietly for 15 seconds, then begin counting each breath and multiplying by four
	d. Instruct the patient to take deep breaths to better visualize respiratory effort

**Table 3:** Pre- and post-test score performance by question (first attempt)

Pre-test (N = 19,412)	Question	Score
	1	86%
	2	76%
Post-test (N = 19,364)	Question	Score
	1	94%
	2	94%
	3	85%
	4	70%

We did not release a formal survey to receive feedback. Due to the widely distributed nature of participants around the world with no centralized organization, we have relied on anecdotal comments solicited via email as our primary means of learner feedback.

A representative quote from an end-user is below:

“... the addition of tele-health (sic) module and assistance in understanding the virtual visit process helped us to be more focused and organized (sic). The technical assistance for conducting virtual visits made it easier to implement the knowledge practically and added to our clinical management skills.”

## Discussion

Though the concepts of telemedicine and virtual visits are not new, acceptance and utilization by both consumers and healthcare providers have been relatively low. As recently as April 2019, a poll showed that patient awareness of telemedicine was only 15% [5]. A survey of physicians in June 2019 showed that only 9% were planning to incorporate telemedicine into their practice [6]. As the benefits of telemedicine were brought to light during the pandemic, many groups around the world rapidly deployed systems to reduce higher risk interactions [7-10]. This led to both medical practitioners and patients being confronted with unfamiliar communication mediums. 

Although other groups have released modules designed to help practitioners use telemedicine effectively, this is a rare study designed for a global audience [3,8,9,11-14]. We did not design a testing instrument to assess the reception of our video module, but we are encouraged by the overall increase in exam scores, which demonstrates an understanding of the basics to maximize a telemedicine interaction. Our demand for quick turnaround precluded our ability to gauge the reception of our educational video with dedicated pre- and post-survey instruments. A future project should include more robust investigations to better understand knowledge acquisition, participant engagement, and acceptability of virtual visit education.

In 2020, the Society for Academic Emergency Medicine held a Telemedicine Consensus Conference, which highlighted the importance of creating educational materials for virtual interactions with patients to improve access to care in low-resource settings. This is precisely the reason for the educational program we created. When first incorporating these platforms, planners should also initiate research protocols in parallel to elucidate ways to maximize the effective deployment of technologies for all patients at all socioeconomic levels from all over the globe. Particular attention should be paid to ensure that future advances do not exacerbate the existing technological gaps, thus leaving vulnerable populations behind.

## Conclusions

Telemedicine and virtual visits have come into vogue after pressure from the COVID-19 pandemic. Because of the limited usage before 2020, educational materials for telemedicine have not been widely developed and disseminated. We created a basic video module to fill this void for our group of 20,000 HCWs distributed around the world. During the creation of our instructional module, we focused on achieving rapid deployment with minimal cost. Based on learner engagement, informal feedback, and participant knowledge gain, we deemed the telemedicine provider education module successful. We anticipate creating future modules dedicated to general best practices with higher production value and generalizability.
