# Correction: Teaching From Afar: Development of a Telemedicine Curriculum for Healthcare Workers in Global Settings

**DOI:** 10.7759/cureus.c68

**Published:** 2022-06-29

**Authors:** Jason T Lowe, Sunny R Patel, Wei D Hao, Jamie Johnston, Abdullah Butt, Matthew Strehlow, Benjamin Lindquist

**Affiliations:** 1 Emergency Medicine/Pediatric Emergency Medicine, Stanford University School of Medicine, Lucile Packard Children's Hospital Stanford, Palo Alto, USA; 2 Emergency Medicine, Weill Cornell Medicine, New York, USA; 3 Emergency Medicine, Stanford University School of Medicine, Palo Alto, USA; 4 Stanford Center for Health Education, Stanford University School of Medicine, Palo Alto, USA; 5 eDoctor, EduCast, Karachi, PAK

This article has been corrected to include Dr. Jamie Johnston among the list of authors. Per the submitting author, "Dr. Lowe led the development of the Telehealth module for the training program which was specifically requested by overseas partners. When submitting the paper, we should have included Dr. Jamie Johnston. I did not identify that she was not included and Dr. Lowe did not have knowledge of all of the behind the scenes work Dr. Johnston was doing."

